# Prognostic significance of regional edema and quantitative assessment of late gadolinium enhancement in patients with acute myocarditis

**DOI:** 10.1186/1532-429X-13-S1-P179

**Published:** 2011-02-02

**Authors:** Emmanuelle Vermes, Helene Childs, Matthias G Friedrich

**Affiliations:** 1 Calgary, calgary, AB, Canada

## Background

Standard diagnostic CMR criteria for acute myocarditis have been proposed (ie Lake Louise criteria) based upon the any 2 of 3 approach which includes the presence of myocardial edema (T2), hyperemia/capillary leakage (early Gd enhancement: EGE) as well as fibrosis (late enhancement: LGE). However, there is a lack of prognostic data using these criteria. The aim of this study was to evaluate the diagnostic CMR criteria for the prediction of functional outcome in patients with myocarditis.

## Methods

We studied 24 patients referred for acute myocarditis during the acute phase and 1 year thereafter. CMR studies included T2 weighted and contrast-enhanced T1 weighted (early and late enhancement) with a quantitative assessment of the regional and LGE.

## Results

In the acute phase, edema (regional and /or global) was present in 67%, EGE in 62.5% and LGE in 54%. Edema %LV (2SD) was: 27.3±7.8; LGE %LV (2SD) was 20.5±14.2. At follow-up, EF significantly increased from 57± 5.5% to 60± 4.5% (p< 0.001). In a univariate analysis, LGE% LV was correlated to the change in EF over 1-year (r=0.704, p=0.04), in a multivariate analysis (linear regression), the combination of LGE%LV and regional edema serves as an independent predictor of an increase of EF (F=5.13, p=0.017)

## Conclusion

In our preliminary experience, the combination of LGE with regional edema was an independent predictor of functional recovery during a 1 year follow-up.

